# Origin of HAV strains responsible for 2016–2017 outbreak among MSM: Viral phylodynamics in Lazio region

**DOI:** 10.1371/journal.pone.0234010

**Published:** 2020-05-29

**Authors:** Claudia Minosse, Francesco Messina, Anna Rosa Garbuglia, Silvia Meschi, Paola Scognamiglio, Maria Rosaria Capobianchi, Giuseppe Ippolito, Simone Lanini

**Affiliations:** National Institute for Infectious Diseases "Lazzaro Spallanzani" I.R.C.C.S., Rome, Italy; Centers for Disease Control and Prevention, UNITED STATES

## Abstract

In Europe HAV infection occurs mainly among specific risk groups, such as consumers of specific food. Sexual transmission of HAV has been demonstrated, particularly among Men-Who-Have-Sex-With-Men (MSM), causing MSM-specific outbreaksin Europe. Here we report a molecular epidemiologic and phylodynamic analysis on HAV sequences in Lazio (central Italy)to identify genetic background and the phylogenetic relations, and test the HAV infection dynamics during a large outbreak through phylodynamic model.Among all HAV sequences found during 2013–2018 in Lazio, low genetic diversity was observed in HAV population in 2016 and 2017, along with high frequenciesVRD_521_2016and RIVM-HAV16-090, suggesting a large expansion event of viral population. The initial expansion of both VRD_521_2016 and RIVM-HAV16-090 clusters dated back to 2012 (95% HPD:2006–2015). During the2016-2017outbreak in Lazio region, the R_e_ peaked around mid-2016, with a value of 1.73 (95% HPD: 1.03–2.37), consistent with incidence trend of AHA cases in Lazio between 2016 and mid-2017. This study showed the magnitude of HAV outbreak in Lazio during 2016–2017, demonstrating the epidemic continuity to MSM-specific outbreak in Europe. The HAV dataset is available on interactive phylodynamic platform https://nextstrain.org to real-time update of future outbreaks.

## Introduction

Hepatitis A virus (HAV) is a globally distributed enteric pathogen known as the cause of acute hepatitis A (AHA), a generally self-limiting infection with an incidence of approximately 1.5 million new cases per year [1]. Mean annual incidence of AHA was very low in 2015 in EU/EEA, (one case per 100.000 persons-year), increasing to 2.4 cases per 100000 population in 2016 [2, 3]. As other enteric human pathogens HAV can be spreadeither indirectly, through contaminated food and water, or directly from person to person, through close personal contacts [4]. Clinical presentation of HAV infections significantly varies with patients’ age: in children under 5 years, HAV infection is mostly asymptomatic, while clinical presentation is more severe in adults, with icteric presentation in up to 70% of cases. Clinical presentation with acute liver failure are rare and mainly associated with older age (>60 year) and liver co-morbidity [5].

The incubation period of HAV infection ranges between 15–50 days (mean 28 days), resolving in 2–8 weeks after activation of humoral and cellular immune response and conferring lifelong immunityagainst HAV [4, 6, 7]. The diagnosis of AHA is usually based on the detection of anti-HAV IgM. HAV RNA can be detected in infected (asymptomatic) patients’ blood and stools [8], since 1–2 weeks before the onset of illness. Viral shedding in feces is very variable and may persist for long time among children and immunocompromised patients including those infected with HIV [9].

In low and very low endemic settings,such as Italy, HAV infections generally occur as sporadic cases in travelers from endemic areas and clusters within either among close (semi-close communities) or among people within special at risk groups [5, 10]. As other enteric pathogens, HAV transmission can be facilitated by oro-anal sex either in homo- or heterosexual partners. However, the oro-anal sex is likely to be more common in the Men-Who-Have-Sex-With-Men (MSM) group. Indeed, since late 1980’s, several large and transnational AHA outbreaks have been associated with same sex intercourse between men [6, 11–13]. In 2016, incidence of AHA abruptly increased across Europe and in particular among Men-Who-Have-Sex-With-Men (MSM). Molecular investigations carried out in different countries suggested that most of the cases where associated with two different HAV genotype IA strains RIVM-HAV16-090 and VRD_521_2016, both associated with outbreaks affecting European MSM communities [6, 14–18]. Particularly, a large outbreak dominated by few strains occurredbetween 2016 and 2017 in Lazio, an administrative region in central Italy whose main city is Rome [17]. In this paper we used the molecular characterization, phylogenetic reconstruction and phylodynamic analysis of the recent genotype IA HAV strains circulating in Lazio as a tool for modelingtransmission pathways and improve outbreak surveillance.

### Short summary of AHA outbreak

MSM-specific AHA outbreak in Europewas between June 2016 and December 2017. The number of outbreak-confirmed cases reported in 24 EU/EEA countries (Austria, Belgium, Croatia, Denmark, Estonia, Finland, France, Germany, Greece, Ireland, Italy, Latvia, Lithuania, Luxembourg, Malta, the Netherlands, Norway, Poland, Portugal, Slovenia, Slovakia, Spain, Sweden and the United Kingdom) was 4 475 (7 September 2018), identified by laboratory testing. The highest male/female ratio (M/F) was 4.8, reported from March to May 2017 in 24 EU/EEA countries. Outbreak-confirmed cases hold one of the three HAV genotype IA outbreak strains (VRD_521_2016; RIVM-HAV16-090; and V16-25801) based on overlapping fragments at the VP1-2a region. No other strains have been reported to be widely circulating among MSM [19]. In Italy between 1 June 2016 and 31 March 2017 the AHA incidence was more than doubled, compared with same period in 2012–2016: in fact, 976 average cases value was reported in 2016–2017, while 421 average cases value was described in 2012–2016. The M/F ratio in Italy was 8.4 during outbreak period against 1.5 in 2012–2016 [20]. In Lazio between 1 January 2016 and 31 March 2017, 87.52% were men, 6.24% women, and 6.24% children with the median age 33 years [21].

## Materials and methods

### Samples collection and molecular investigation

The samples were collected in the context of the AHA surveillance program to monitor possible AHA outbreaks, especially among young MSM [6, 17, 18] and to highlight changing patterns of genetic diversity of HAV isolates in Lazio region between 2013 and 2018. The surveillance and control activities of the 10 local heath units operating in the region has been coordinated by Lazio Regional Service for the Epidemiology and Control for Infectious Diseases (Servizio Regionale per l'Epidemiologia e la Sorvegliaza delle Malattie Infettive—SERESMI), that has been established since 2015 at the National Institute for Infectious Diseases "Lazzaro Spallanzani" (INMI) in Rome. This work was supported by funds from the Italian Ministry of Health (Ricerca Corrente “Linea 1” and Ricerca Finalizzata) and the European Union’s Horizon 2020 research and innovation program, European Virus Archive (EVAg, grant no. 653316).

The samples from all AHA cases are submitted for confirmatory testing to the regional reference laboratory, established at the Laboratory of Virology of INMI. Confirmatory testing includes HAV-specific antibody evaluation and HAV RNA detection on all submitted samples. A representative sample of either serum or stool specimens with detectable HAV RNA are sequenced for molecular epidemiology assessment. Anti-HAV IgG and IgM detection was performed with Architect system (Abbott Diagnostics, Abbott Park, IL, USA). RNA was extracted from serum or stool samples, depending on available material, using QIASYMPHONY automated instrument (QIAGEN, Hilden, Germany). RNA was eluted in 60 microliters elution buffer, 5 microliters of them was reverse transcribed, and cDNA was amplified using specific primers for VP1X2A junction [8]. Particularly, reverse transcription and first–round PCR were conducted using OneStep RT-PCR Kit (QIAGEN, Hilen, Germany), in 50 μL reaction mixture containing 5 μL of extracted RNA, 10 μL of 5xBuffer, 2 μL of dNTPs (10mM) and 2 μL of OneStep enzyme. Final concentrations of primers (labelled HAV6.1_codehop and HAV10_codehop) were 0.6 μM. Reverse transcription was performed at 50°C for 30’ followed by the PCR reaction. Thermal cycling conditions were as follows: 15 min at 95°C, followed by 35 cycles of 30 s at 95°C, 30 s at 42°C, and 45 s at 72°C, with a final step of 7’ at 72°C. The second round PCR was conducted using AmpliTaq Gold™ DNA Polymerase (Applied Biosystems, Forster City, CA, USA), in 50 μL reaction mixture containing 1 μL of first round of PCR, 5 μL of 10xBuffer, 4 μL of MgCl2, 1 μL of dNTPs (10mM) and 0.3 μL of enzyme. Final concentrations of primers (labelled HAV8.2_codehop and HAV11_codehop) were 0.5 μM. Thermal cycling conditions of second round of PCR reaction were as follows: 5 min at 95°C, followed by 40 cycles of 30 s at 95°C, 20 s at 60°C, and 15 s at 72°C, with a final step of 7’ at 72°C. Positive and negative controls, which contained standardized viral RNA extracts and nuclease-free water, respectively, were included in each RT-PCR assay. The resulting amplicons (of 518 nt) were sequenced by automated Sanger sequencing with Prism BigDye (Applied Biosystems, Forster City, CA, USA) in an ABI3100 DNA Sequencer. HAV database with 246 sequences obtained at the regional reference laboratory, including 222 sequences from January 2016 to December 2018 and 24 sequences from the 2013–2015 years, was established for the present analysis.

### Statistical inference of HAV sequence data

The 459 nt sequences encompassing the VP1X2A junction region of HAV genome was aligned by CLUSTALW program for multiple sequence alignment [8], which at first was used to build a maximum-likelihood method phylogenetic tree, implemented in MEGAX software [22]. To assess the significance of the nodes, bootstrap analysis with 10,000 replicates was performed (>80% considered significant). In the phylogenetic tree, all the HAV sequences obtained from cases referred to the Laboratory since 2013 were included. Reference sequences, retrieved from GenBank, were included as well. In addition, the sequences of four strains, reported to be associated with current MSM-linked epidemic clusters in other European countries, were included in the phylogenetic analysis: VRD_521_2016, first described in United Kingdom, RIVM-HAV16-090 and RIVM-HAV16-069 first described in Netherlands, and V16_25801, first reported in Germany [6, 18, 23]. For this dataset, the best fitting nucleotide substitution model was selected by *modelTest* function, implemented by R packages *ape* and *phangorn* [24, 25]. By this tool, the General time reversible model plus Gamma distributed rates among sites [26] (GTR+G with shape parameter = 0.25) was selected by the highest values of loglikelihood (logLik) and Akaike Information Criterion (AIC) (best model GTR+G: max logLik = -3111.83 and AIC = 7287.667).

To determine the population genetic features of recent HAV outbreak in Lazio, the entire dataset was split by year in four viral populations (2013–2015; 2016; 2017; 2018). Standard diversity and molecular indices, i.e. number of unique variant sequences (K), number of polymorphic sites (S) and gene diversity (H), were calculated for viral populations of each year. Gene diversity (H) is defined as H = $\frac{n(1-\Sigma x_{i}^{2})}{\left(n-1\right)}$, where x_i_ is the frequency unique variant sequences in the sample and *n* is sample size [27]. It indicates the degree of diversity among sequences into viral population and its values is between 0 and 1, where 0 is absent of diversity among sequences and 1 is absent of equality among sequences. Finally, Tajima's D was used to determine the effect of selection pressure of each viral population per each year. It is defined as D = $\frac{\theta_{\pi }-\theta_{S}}{\sqrt{Var(\theta_{\pi }-\theta_{S})}}$ where θ_π_ is a parameter based on the mean number of pairwise differences among unique sequences, while θ_S_ is based on the number of polymorphic sites (segregating) in the sample [28]. For each year, the genetic diversity indices of viral populations were estimated using the software Arlequin 3.5 [29]. The significance was tested through 10,000 permutations, and unique variant sequences for each viral population were used as analysis dataset, accounting for 48 sequences overall and including genotypes IIIA, IB and IA. This approach determines differences in genetic diversity among the four viral populations, arising from variation in effective population size. The synonymous mutations per synonymous site (dS), the non-synonymous mutations per non-synonymous site (dN) and the overall average dN/dS ratio of all viral sequences from each viral population per year were calculated using MEGAX, using Nei and Gojobori method [30]. dN/dS>1 means the trait is not under positive selection, while dN/dS<1 is under strong positive selection. If dN/dS = 1, it was selective neutrality condition [31]. Recombination analysis on VP1X2A junction sequences was carried out by RDP4 software [32].

The pairwise genetic distances Fst was used to evaluate the genetic variances between years of infection, through 10,000 permutations and with p<0.05 [33–35]. Fst represent genetic distances between samples and it is estimated by $Fst=1-\frac{\pi_{Within}}{\pi_{Between}}$ where π_Within_ is mean number of pairwise differences among viral sequences within sample, while π_Between_ is between two samples [36].

### Phylodynamic analysis

For phylogeny analyses the dataset, containing 48 unique HAV variant sequences representing the HAV strains circulating in Lazio in the study period (2013–2018), was enlarged, including 101 additional HAV worldwide unique sequences retrieved from GenBank, with exhaustive epidemiological and geographical information.

At first, this dataset was analyzed through web-based Nextstrain tool, which uses a augur pipeline [37], for reconstruction of viral phylogeny [38, 39]. The augur pipeline was run using the aligner MAFFT [40], the tree-builder [41], and the phylodynamic package TreeTime [42]. The repository detailing the analysis pipeline is available at https://github.com/INMIbioinfo/HAV_VP1-2A. The resulting analysis is visualized by auspice and is publicly available at https://nextstrain.org/community/INMIbioinfo/HAV_VP1-2A. AY644670.1 sequences (reference for IIB genotype) were excluded from the phylodynamic analysis because it deviated more than 4 interquartile ranges from the molecular clock regression.

To explore phylogenetic relationship within HAV genotype IA, a maximum-likelihood method phylogenetic tree was built, using MEGAX software [22], while to test clock signal in genotype IA database was used TempEst v1.5 [43].

To estimate demographic history of HAV genotype IA infections, the best fitting nucleotide substitution model was identified as the GTR+G by all model selection methods (best model GTR+G: max logLik = -3138.714 and AIC = 6561.428), through *modelTest* function of R packages *ape* and *phangorn* [24, 25]. Bayesian phylogenetic trees were inferred using Bayesian Markov chain Monte Carlo (MCMC) approach, available in BEAST v1.10.4 [21]. Independent MCMC runs were carried out for strict and relaxed clock models, along with the following coalescent priors: constant population size, exponential growth and nonparametric Bayesian Skyline plot (BSP) [44, 45]. By BEASTv1.10.4 function, marginal likelihoods estimates for each demographic model were obtained through path sampling and stepping stone analyses, and the best-fitting model for the dataset was determined by calculating the Bayes factors (BF) [46, 47]. By this approach, a pair wise comparison of models can be carried out, in which Bayes factor between two models is a ratio of two marginal likelihoods obtained for the two models, M_0_ and M_1_: 2lnBF<0: negative evidence for M_0_ (supports M_1_); 0.0–2.2: not worth more than a base mention; 2.2–6.0: positive evidence; 6–10: strong evidence; >10 very strong evidence [48]. Chains were conducted for at least 100x10^6^generations, and sampled every 10,000 steps for each molecular clock model. The MCMC convergence was assessed by calculating the ESS for each parameter, accepting values >250. Maximum Clade Credibility trees were obtained from the trees’ posterior distributions with Tree-Annotator software v 1.10.4 [21]. Statistical support for specific monophyletic clades was assessed by calculating the posterior probability (pp). Bayesian skyline plot (BSP) was inferred by BEAST v1.10.4 and plotted by Tracer 1.7.1 [49]. BSP shows virus effective population size (Ne) trend, i.e. the effective number of viral genomes contributing to new infections, respect to time (years), assuming that the coalescent events on a small random sample can be an approximation of the large population dynamics [50, 51]. The variation of the transmissibility of HAV strains spreading in Lazio region along the study period was estimated on the basis of the effective reproduction number (R_e_), calculated with the birth–death skyline (BDSKY) approach. The birth–death model allow to quantify an epidemiological parameter to estimate the transmissibility of infectious disease, namely the effective reproduction number (R_e_), which is given by this ratio: R = λ/δ, in which each infected individual may transmit with a rate λ and eventually becomes uninfectious with a rate δ, which is sum of rates of death/recovery and sampling, μ and ψ, respectively [52]. R_e_ is defined as the average number of potential secondary infections from an infected individual in the presence of herd-immunity at each time point during the outbreak [52, 53]. R_e_>1 indicates increasing number of cases, and a potential epidemic, while R_e_<1 indicates that the number of cases decreases with each generation, and if the decrease is maintained the epidemic will be eliminated. The method is implemented as an add-on BDSKY to the BEAST v2.5.2 software framework [54]. Chains were conducted for at least 100x10^6^ generations, and sampled every 10,000 steps and General Time Reversible model plus Gamma distributed rates among sites (GTR+G) was used under strict clock model.

### Ethical approval

According to REGULATION (EU) 2016/679 OF THE EUROPEAN PARLIAMENT AND OF THE COUNCIL of 27 April 2016 on the protection of natural persons with regard to the processing of personal data and on the free movement of such data, and repealing Directive 95/46/EC (General Data Protection Regulation). Informed consent was not obtained from the participants because of the public health emergency during an infectious disease outbreak. All data contained in the manuscript were obtained during the epidemiological investigation as an institutional duty of the Lazio Regional Health Authority, in order to identify/contain the ongoing outbreak, to provide recommendations on control measures and to avert complications in infected subjects. The approval of the National Institute for Infectious Diseases Spallanzani’s Institutional Review Board was not required for the same reasons. Patients never underwent individual intervention for the purposes of this study but only according to their needs and clinical judgment. Data have been analysed anonymously.

### Limitations

The main limitation of our study is the use of a short HAV genome region (VP1X2A junction, 459bp fragment) to study HAV phylodynamics. Although this trait provides more limited genetic information than larger sub-genomic regions or even full-length HAV genome,it was adopted becauseVP1-2A region is the most commonly used to investigate the outbreaks and a large number of VP1-2A sequences are available for comparison in public databases. On the other hand longer sequences linked to recent HAV outbreaks in Europe are generally missing, and this would have represented an even greater limitation for the present phylodynamic study.

## Results

### Trend of HAV genetic diversity in Lazio over time

To determine the population genetic features of HAV and highlight epidemic trend in Lazio between 2013 and 2018, we estimated viral genetic diversity indices on the entire database of HAV VP1X2A junction sequences from Lazio, using summary statistics indices (Table 1). The molecular diversity values were computed considering four time intervals (2013–2015; 2016; 2017 and 2018); the genetic variability parameters included were number of variant sequences (K), number of polymorphic sites in the alignment (S) and gene diversity (H), which indicate the degree of diversity among sequences in viral population. The expansion of viral population was tested by Tajima's D.The results indicated a decrease of all three parameters of genetic variability between 2016 and 2017, followed by restoration of initial values in 2018. This trend was in keeping with the circulation of epidemic HAV strains between January 2016 and March 2017, as suggested by surveillance data and by previous molecular investigation [17]. Moreover, Tajima's D values for viral populations in 2016 (D = −1.76585) and 2017 (D = -1.57359) were significantly less than zero (p < 0.030), suggesting the viral population was involved in an expansion event in these years. Average overall dN/dS ratio resulted<1 for all periods, indicating absence of positive selection, at least in the 459 bp VP1X2A junction region. Moreover a recombination analysis among all sequences, including reference strains, highlighted no evidence of recombination event. To quantify genetic similarity among HAV viral populations over time and determine the extent of genetic variability, Fst pairwise distance was evaluated (Table 2). The lowest Fst value was observed for 2016 vs 2017 (0.02322, p = 0.030), confirming a unique large epidemic event with genetic continuity in these years. This was in line with the low gene diversity values in 2016 and 2017 (Table 1), and with the presence of a largely dominant strain, i.e. VRD_521_2016, accounting for 80% and 69% of all sequences in 2016 and 2017, respectively (S1 Table). The second dominant strain was RIVM-HAV16-090, accounting for 22% and 8% of all sequences in 2016 and 2017.

**Table 1:** Indices of genetic diversity of HAV sequences (VP1-2A region) from Lazio region, grouped according to the year of sampling (2013–2015 2016, 2017, 2018). N: sample size; K n. of variant sequences; S: n. of polymorphic sites; H: gene diversity; Tajima's D:test to highlight in population any deviation from neutral evolution; dN/dS: ratio of non-synonymous mutations per non-synonymous site (dN) and synonymous mutations per synonymous site (dS) of all viral sequences from each viral population in years.

Years	N	K	K/N	S	H	Tajima's D	P value	dN/dS
2018	37	14	0.37	135	0.8483 +/- 0.0425	-0.48477	0.35920	0.023
2017	101	17	0.16	73	0.5127 +/- 0.0602	-1.57359	0.02750	0.025
2016	84	14	0.16	76	0.3643+/- 0.0684	-1.76585	0.01160	0.013
(2013–2015)	24	12	0.5	126	0.8696 +/- 0.0547	0.00145	0.57150	0.038

**Table 2:** Pairwise comparison of genetic diversity of HAV sequences (VP1X2A junction) from Lazio region in different years. Fst value represents the genetic distance between viral population in the years 2013–2015 2016, 2017, and 2018. Statistical significance of each pairwise comparison is in brackets.

Fst (p value)	2018	2017	2016	2013–2015
2018	*			
2017	0.24341 (0.0000)	*		
2016	0.27886 (0.0000)	0.02322 (0.0318)	*	
2013–2015	0.14788 (0.0000)	0.51566 (0.0000)	0.50019 (0.0000)	*

**Fig 1:**
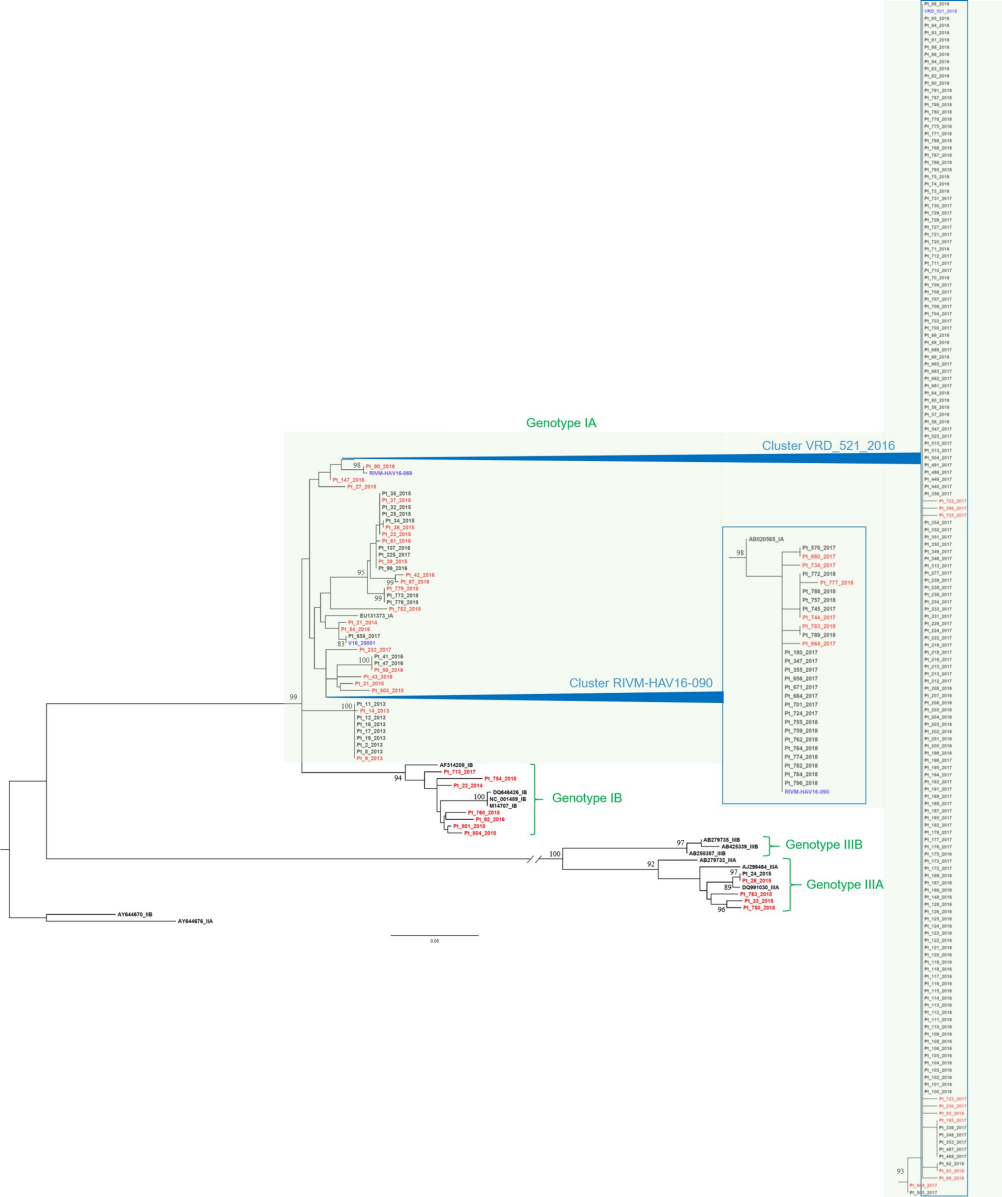
Maximum-likelihood phylogenetic analysis of all the sequences obtained until October 2018 from Lazio region. Phylogenetic Maximum-Likelihood tree, built with a total of 246 459 nt-long sequences encompassing the VP1X2A junction region of HAV genome, based on the maximum-likelihood method with the General Time Reversible model + G. All the sequences obtained until October 2018 from Lazio region are included. Moreover, the tree includes 16 reference sequences from GenBank (genotype IA: EU131373; AB020565.1; genotype IB: M14707; DQ646426; NC001489; AF314208; genotype IIA: AY644676; genotype IIB: AY644670; genotype IIIA: AJ299464; DQ991030; AB279733; genotype IIIB: AB279735; AB425339; AB258387), and four sequences (VRD_521_2016 and RIVM-HAV16-90, RIVM-HAV16-69 and V16_25801, in blue) associated with epidemic clusters among MSM. The bar represents the genetic distance (substitution per nucleotide position). Bootstrap analysis with 10,000 replicates was performed to assess the significance of the nodes; values greater than 80.

Preliminary phylogenetic analysis of all HAV sequences database from Lazio underwent an exploratory ML phylogenetic analysis. The analysis allowed to split the main genotypes, underlined by names in green. In genotype IA (light green background), two large clusters associated with strains VRD_521_2016 and RIVM-HAV16-090 were shown (Fig 1). The unique variant sequences were highlighted in red. The VRD_521_2016 cluster, firstly identified in Lazio at the end of August 2016, is the largest cluster of entire tree, with 165 sequence and 11 unique variant sequences and a statistically significant node (93% bootstrap value). RIVM-HAV16-090 cluster included 28 sequences (six unique) found between 2017 and 2018, with a statistically significant node (98% bootstrap value).

Five sequences from 2017–2018 cases put on HAV genotype IB, while two sequence from 2018 cases fell into HAV genotype IIIA. Overall, VRD_521_2016 and RIVM-HAV16-090 have contributed strongly to AHA epidemic event in that period, while V16_25801 was poorly represented, and RIVM-HAV16-069 did not contribute [17].

A similar analysis based on the HAV sequences from Lazio region and sequences from another European country experiencing a contemporary AHA outbreak [55], based on a shorter (319 bp) overlapping VP1X2A junction region, showed that, indeed, the same HAV IA epidemic strains, i.e. VRD_521_2016,RIVM-HAV16-090 were mainly involved in the contemporary outbreak in Italy, Lazio and Spain, Barcelona (S3 Fig).

### HAV phylogeny reconstruction and population dynamics

**Fig 2:**
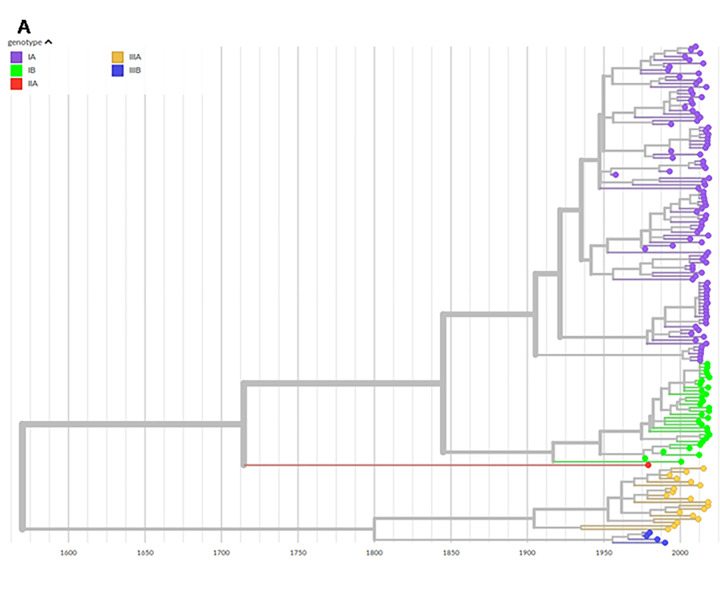
Phylogenetic analysis of HAV genotypes strains. The panel A shows a phylogenetic tree as obtained by Nextstrain, containing VP1X2A junction sequences of HAV genome from Lazio region, Europe and worldwide. All information about geographic origin and year of isolation for each strain is reported in S2 Table. In this tree the sequences were labelled by HAV genotype.

To identify the geographical origin and reconstruct the phylogeny of HAV on time scale, 48 unique variant VP1X2A junction sequences from Lazio were selected from initial database of 246 HAV VP1-2A sequences. The new HAV database was enriched by RIVM-HAV16-069 strain and 101 HAV sequences available on GenBank. At first, an explorative analysis of temporal and phylogenetic relationship was done using the web-based Nextstrain tool (https://nextstrain.org/community/INMIbioinfo/HAV_VP1-2A), which allows users to interactively explore the dataset. A positive correlation between root-to-tip and time estimated clock rate 3.89*10^-4^subst/site/year, while the trees howed main genotypes as well-defined phylogenetic groups (Fig 2). When the same sequences were labelled by country, two recent clusters in IA genotype,containing sequences only from Lazio region, were marked by number 1 and 2, clustering along with RIVM-HAV16-090 and VRD_521_2016 strains, respectively (S1 Fig). VRD_521_2016 and RIVM-HAV16-090 clusters were datedto 2009 (CI: 1999–2013) and 2006 (CI: 2002–2015), respectively.

**Fig 3:**
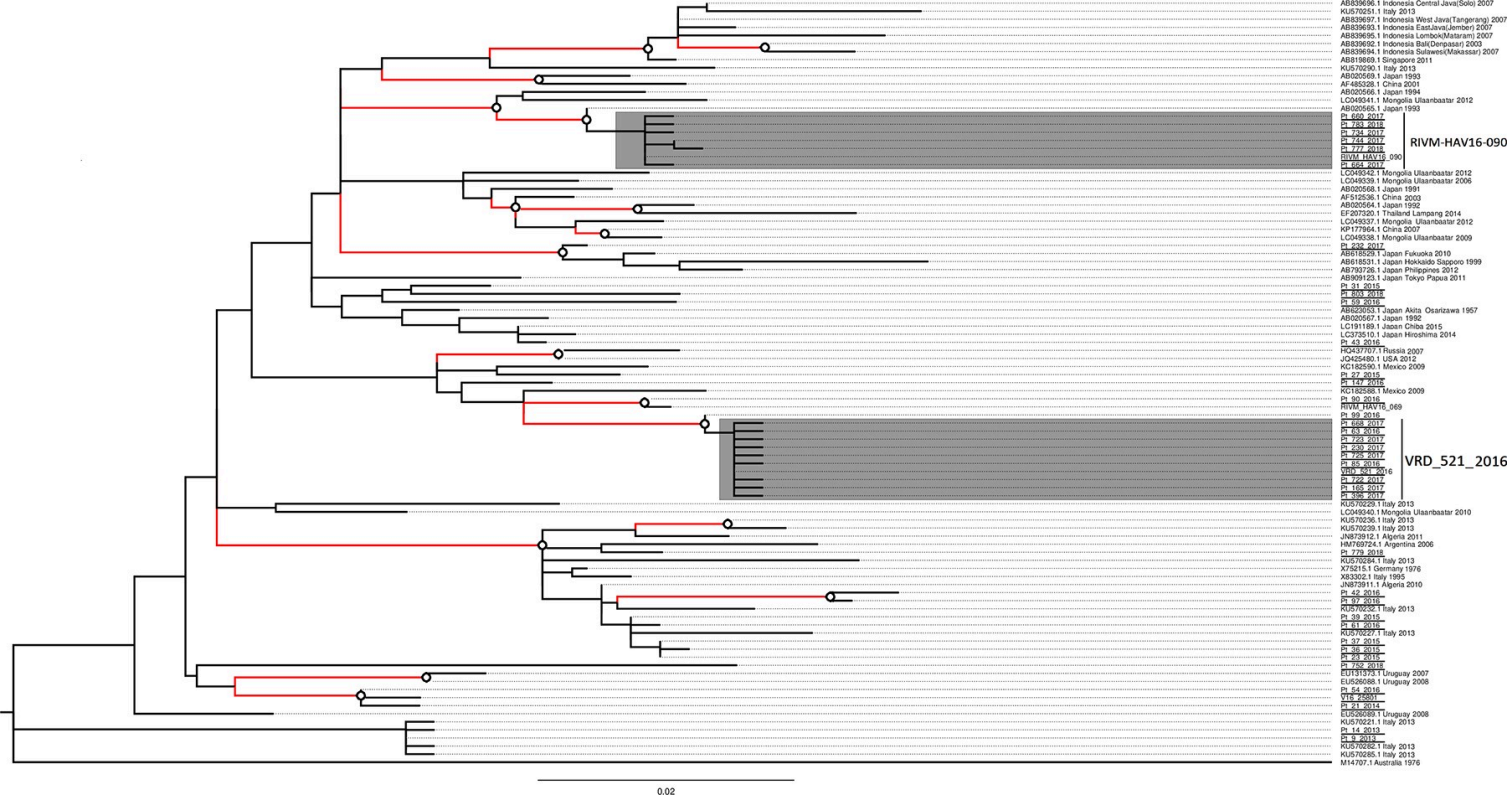
Maximum likelihood tree of genotype IA dataset. The evolutionary history of HAV was inferred on VP1X2A junction region of HAV genome, using the Maximum Likelihood method with GTR+G mutation model. The tree is drawn to scale, with branch lengths measured in the number of substitutions per site. Evolutionary analyses were conducted in MEGA X [22]. Bootstrap values are greater than 80% (○) and the nodes are marked by coloured braches. Branches in grey represent VRD_521_2016 and RIVM-HAV16-090 clusters. All unique sequences, collected until October 2018 from Lazio region and classified as genotype IA, are included and sample names are underlined in the tree.

To describe phylogeny of VRD_521_2016 and RIVM-HAV16-090 clusters more thoroughly, ML tree was built using only genotype IA sequences, using IB reference M14707.1 as an outgroup. Phylogenetic relationships were defined through> 0.8 bootstrap values (Fig 3) and the clock signal in genotype IA data was also tested using root-to-tip regression approach, which allow to explore the association between genetic divergence through time and sampling dates [43]. In this data set a positive correlation between root-to-tip divergence and sampling time was showed, with clock rate estimated of 1.66*10^-4^subst/site/year.

Moreover, VRD_521_2016 and RIVM-HAV16-090 clusters were highlighted and limited by statistically significant nodes.

**Fig 4:**
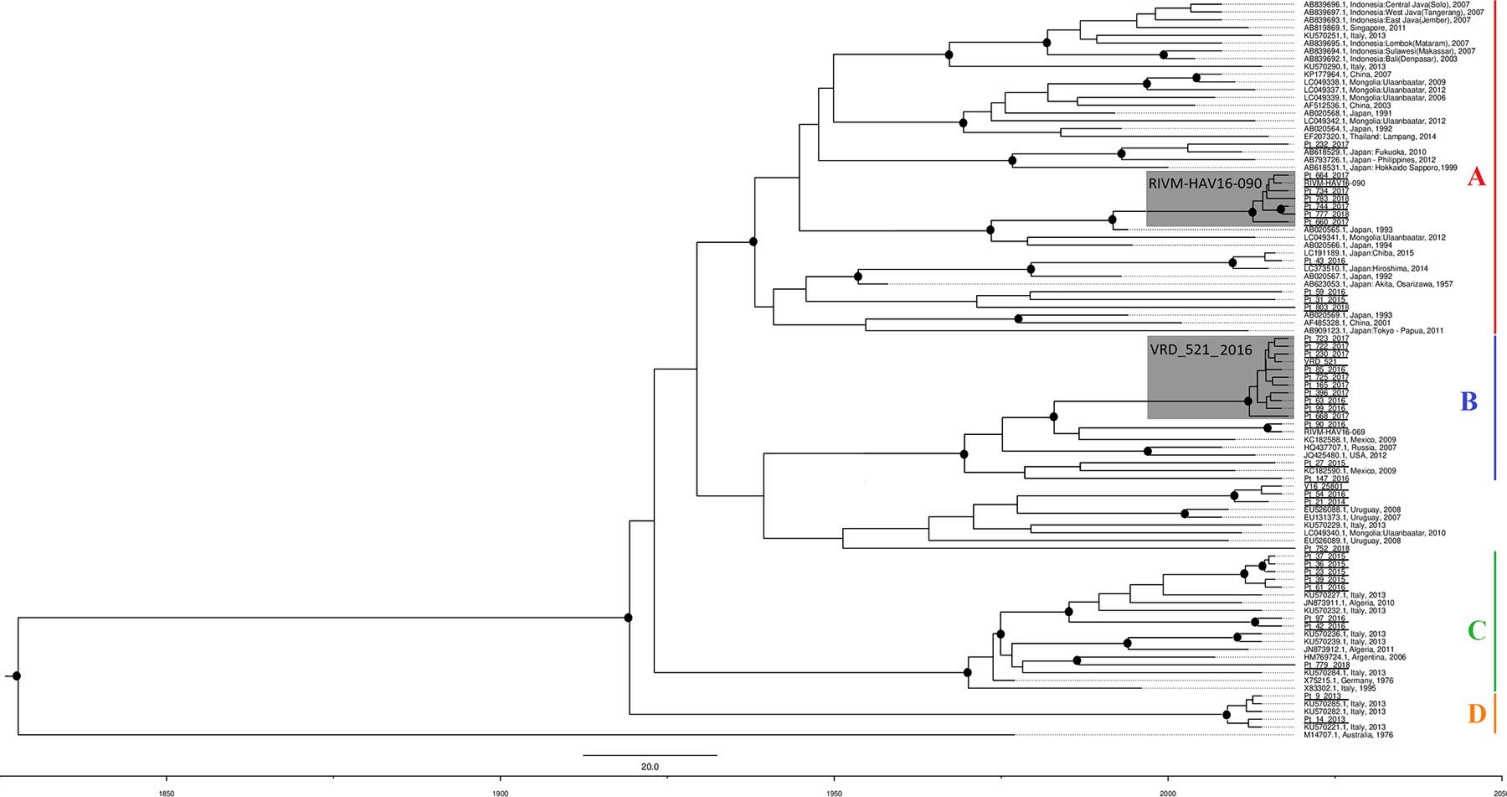
Bayesian Phylogenetic tree. Bayesian Maximum Credibility (Bayesian MCC) phylogenetic tree, built on 46 459 nt-long VP1X2A junction sequences of genotype IA HAV genome. The model used was: mutation model General Time Reversible + G, strict clock model and Bayesian skyline demographic model (2lnBF > 10 for every comparison), tested through MCMC for at least 100*10^6^ generations. All unique sequences, collected until October 2018 from Lazio region and classified as genotype IA, are included and sample names are underlined in the tree. The bar represents time coalescent in years and posterior probability values are greater than 80% (●). The tree also includes 53 sequences from GenBank (AB020564.1, AB020566.1, AB020567.1, AB020568.1, AB020569.1, AB618529.1, AB618531.1, AB623053.1, AB793726.1, AB819869.1, AB839692.1, AB839693.1, AB839694.1, AB839695.1, AB839696.1, AB839697.1, AB909123.1, AF485328.1, AF512536.1, EF207320.1, EU131373.1, EU526088.1, EU526089.1, HM769724.1, HQ437707.1, JN873911.1, JN873912.1, JQ425480.1, KC182588.1, KC182590.1, KP177964.1, KU570221.1, KU570227.1, KU570229.1, KU570232.1, KU570236.1, KU570239.1, KU570251.1, KU570282.1, KU570284.1, KU570285.1, KU570290.1, LC049337.1, LC049338.1, LC049339.1, LC049340.1, LC049341.1, LC049342.1, LC191189.1, LC373510.1, M14707.1, X75215.1, X83302.1), and the 4 sequences (VRD_521_2016 and RIVM-HAV16-90, RIVM-HAV16-69 and V16_25801). The bar represents time coalescent in years. Coloured lines on the tree marked clades in genotype IA, while the grey background highlights MSM clusters with RIVM-HAV16-090 and VRD_521_2016.

Bayesian phylogenetic approach was applied to genotype IA clade to allow dating of VRD_521_2016 and RIVM-HAV16-090 clusters more specifically. The Bayesian skyline demographic model with a strict molecular clock was selected as the most appropriate to describe the evolutionary history of enter HAV phylogeny (2lnBF > 10 for every comparison). The presence of both clusters was showed by Bayesian phylogenetic tree (Bayesian MCC), where was also confirmed by posterior probability > 0.80 (Fig 4). The evolutionary rate estimated on the this dataset was 4.41*10^−4^ subst/site/year (95% HPD:3.28*10^−4^–5.4*10^−4^), consistent with both previous root-to-tip analyses and estimated rates of HAV genotype IA [56–60]. The most common recent ancestor (tMRCA) in the root of the tree corresponded to 1826 (95% HPD:1754–1881). Bayesian MCC tree showed four main clades into genotype IA: A (red), B (blue), C (green) and D (orange).

Clade A included 12 sequences from Lazio sampled in 2016–2018 along with 29 sequences from East Asia and the root node was dated back to 1937 (95% HPD:1920–1950). In this clade RIVM-HAV16-090 cluster was clearly inserted, dated to 2012 (95% HPD:2006–2015).

Clade B contain 19 sequences with common node dated back to 1972 (95% HPD:1960–1983): 14 from Lazio region, sampled between 2015–2017, three sequences from North America, one from Russia and two strains associated with MSM outbreak, VRD_521_2016 and RIVM-HAV16-069. Cluster VRD_521_2016 contains 12 sequences sampled in 2016–2017, with an expansion in 2012 (95% HPD:2006–2015), probably due to the outbreak in MSM. Clade C has 18 sequences, 14 from Italy (eight from Lazio), two from Algeria, one from Germany and one from Argentina, in which main node was dated back to 1969 (95% HPD:1956–1976). In this clade, a cluster of sequences sampled in 2015 in Lazio was already associated with food consumption in this region and it was arose in 2007 (95% HPD:2000–2012) [17]. At last, the Cluster D was the smallest group (only 5 sequences sampled in 2013), which would be separated from main group of genotype IA in 1918 (95% HPD:1887–1942) and expanded in 2008 (95% HPD:2000–2012).

The Bayesian skyline plots of HAV effective population size were built by genotype IA. A growth by degrees for genotypes IA infection number was estimated with two exponential growth events around 1940 and 1975, up to the peak around 1998 and then a strong decreasing after 2005 (S2 Fig). The lowering of infection number after 2005 could be consistent but also significant reducing hepatitis A incidence rates by universal immunization of children with hepatitis A vaccine from mid-1990s [61].

### Estimating of effective reproduction number (R_e_) for AHA outbreak among MSM in Lazio

As the dataset of HAV VP1X2A junction sequences from Lazio set up for this study closely represented the incidence trends of AHA cases in Lazio between 2013 and 2018 [17], it was a good chance to estimate the effective reproduction number (R_e_) through the birth-death skyline (BDSKY) model [52, 53]. For BDSKY it was necessary to choose prior epidemic parameters, both constant and changing across intervals through time in order to reflect natural trend of an outbreak [62]. BDSKY prior parameters for HAV outbreak were followed: one interval for the become uninfectious rate was set with a prior distribution to Uni (5, 120), corresponding to time period, in which AHA patient is infectious, or 3 days—73 days [4, 7, 63]; one interval for the sampling probability was set with the prior distribution to Beta (1, 9999), the sampling probability before the first sample was set to 0; for effective reproduction number (R_e_) ten equidistant intervals between the root and the last sample was chosen with LN (2, 1.25) prior distribution, 0 as the lower and 50 as the upper bound; for the origin parameter in years, the prior was set in Uni (7,8), because the outbreak starting was well-know.

**Fig 5:**
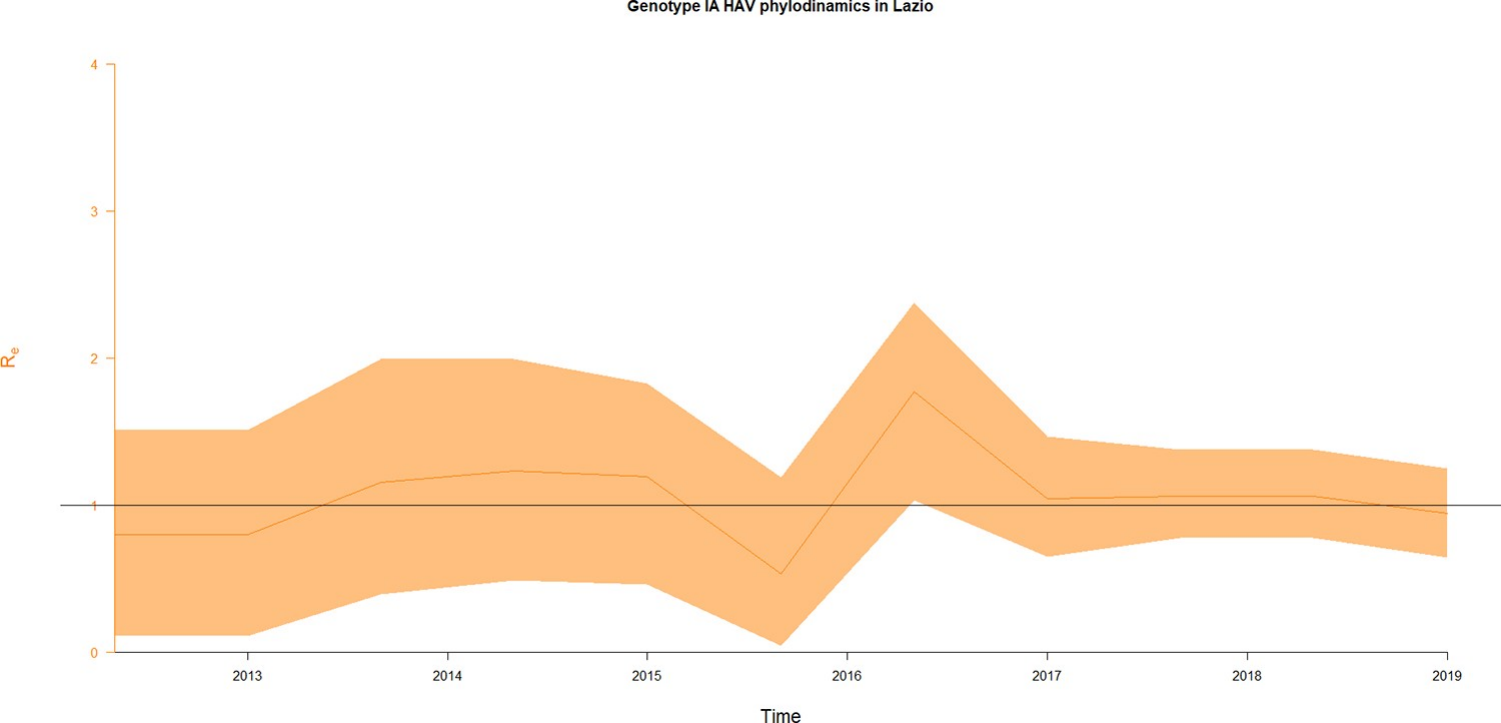
Birth–death skyline plot of HAV in Lazio. Plot of Birth–death skyline on the entire dataset of every unique VP1X2A junction sequences, based on data from Lazio region in the 2013–2018 interval. The orange line represents the median estimates for the effective reproduction number (R_e_), while the yellow area represents its HPD intervals.

For BDSKY model on HAV dataset from Lazio, R_e_ trend between end-2013 and end-2018 is shown in Fig 5. The becoming uninfectious rate δ was estimated to be 6.04 per lineage per year, which means an infectious period of 57 days.

R_e_ showed values higher than 1 during 2013 and 2014 (1.23–1.17), declined in 2015 and then increased steeply in early 2016, reaching a peak value of 1.73 in mid-2016 (95% HPD: 1.03–2.37). After the 2016 peak, R_e_ declined to reach a plateau of 1 around early 2017, which was maintained up to mid-2018.

## Discussion

The WHO calls for elimination of viral hepatitis as a public health threat by 2030 [64]. Though HAV is not associated with chronic infections, the large number of incident cases and the propensity of this virus to produce long-lasting epidemic may significantly impact on individual well being, producing relevant direct and indirect costs at local level. Strategy for optimizing the control of HAV should be tailored on local epidemic profile: in settings where HAV is low and very low endemic, a prompt identification of outbreak within special risk groups is a critical step for scaling up interventions to stop the transmission and avoiding large outbreak. Indeed, special groups at risk living in low endemic settings, represent the ideal setting for explosive outbreak due to the significant prevalence of behavior facilitating HAV transmission and low level of immunity.

Starting from real epidemiological data, we applied phylodynamic methods to assess the behavior of HAV during a large outbreak of AHA, which occurred in Lazio between 2016 and 2017, mainly affecting the MSM population as a model of special risk group. The observed low genetic diversity among HAV variants during the outbreak, along with the significant prevalence two strains only (VRD_521_2016and RIVM-HAV16-090) suggested that the trigger of the expansion was mainly associated with host dependent factors (e.g. super spreading behaviours among a largely susceptible population) rather than virus dependent factors (e.g. positive selection of hyper-infective strain).

This hypothesis is consistent with the observation that also in other AHA outbreaks, infections were mainly due to few strains and epidemiologically linked to hosts’ special condition or lifestyle [8, 17, 65]. For example, HIV sero-status and special behaviours facilitating the infection trigged a large outbreak in Rome between 2008 and 2010 among HIV-infected MSM [14, 16, 66]. Also in this circumstance the whole outbreak was characterized by few HAV strains, without special genetic mutation, already associated with MSM outbreaks.

The topology of VRD_521_2016 and RIVM-HAV16-090 clusters, obtained in all of phylogenetic analyses, showed a “‘star-like” shape, with external branches longer than the internal ones, and is typical of rapid expansion of a virus in a target population [67]. The analysis of coalescent events from Bayesian MCC tree suggests for both clusters an origin dating back to 2012 (95% HPD: 2006–2015), which was consistent with Nextstrain dating. This dating could indicate a latency period, in which outbreak strains could silently circulate in MSM community, showing a range only after EuroPride 2016, which took place in Amsterdam in July/August 2016 [18]. The explosive expansion of these strains involved many distant countries worldwide, where similar contemporary outbreaks have been described. Early AHA cases linked to MSM outbreaks were found between 2016 in England, Netherland and Germany [18, 23, 68]. Two HAV strains VRD_521_2016 and RIVM-HAV16–090 were found in 1400 cases between June 2016 and May 2017 in Europe, with 56% and 35% respectively, becoming strain markers of this epidemic event [6, 20]. AHA outbreak was not enclosed in Europe: cases with epidemic strain markers were found in other extra-EU countries, such as Israel, Brazil and Japan [69–72], while in some cases new local outbreaks among MSM were there [72–74]. In this epidemic event Italy was no exception: since September 2016 Lazio Regional Service for the Epidemiology and Control for Infectious Diseases (SERESMI) has noticed an unexpected increase of AHA cases in Lazio (central Italy), finding a molecular linkage with this AHA outbreak [17], while 15 AHA cases with genotype IA strains linked to same epidemic event were reported in southern Italy [15].

Another important aspect was the possible geographic origin of RIVM-HAV16-090 cluster: the closest relative of this cluster was a sequence (AB020565) collected in Japan in 1993 [75], suggesting a possible origin of the RIVM-HAV16-090 in Far Eastern Asia.

Finally, we applied bayesian phylodynamic approach to HAVmolecular data, through birth–death skyline model (BDSKY), to estimate the R_e_ during the 2016–2017 outbreak.

The same model has already been used to model changes of R_e_ over time to realistically describe the epidemic dynamics of a viral infection of public health importance. For instance, peak of transmissibility (R_e_ ~ 2) modelled through BDSKY in a Dengue virus outbreak in urban area of Brazil in March 2012 coincided with the observed peak in Dengue virus detection in blood-donor banks [76]; similarly, for Zika Virus BDSKY allowed to forecast an increase of R_e_ in Florida (USA) in June 2016, before the first reports from Florida Department of Health [62].

In the Lazio AHA epidemic dynamics, the R_e_ distribution was featured by values constantly higher than 1 (1.23–1.17) in 2013–2014 and a peak around mid-2016 with R_e_ 1.73 (95% HPD:1.03–2.37). Both signals were consistent with the incidence trends of AHA cases in Lazio in mid-2013 with 30 AHA cases, which represented more than 2 times the total of AHA in whole year 2012 [65], and between 2016 and mid-2017, where the incidence of AHA increased from less than 2 (January-August 2016) to 46.9 (March 2017) cases per 100,000 resident-years in Rome [17]. The R_e_ estimated for AHA outbreak among MSM in Lazio is consistent with R_e_ estimated by a different bayesian model for household transmission events during the same epidemic, where the resulting R_e_ was 1.63 (95% CI: 1.35–1.94) [77]. Moreover, many epidemic studies about AHA reported epidemic dynamics similar to this outbreak: in MSM population in Sydney between 1991 and 1992 R_0_was 3.3 (95% PI: 1.71–3.67) [63]; in England in 1986–1987 the R_0_ estimated by AHA average annual incidence in children was 1.6–2.2 [78]; in AHA outbreak at an elementary school in China in 2011 R_0_ was estimated between 2.1 and 2.8 (95% CI: 1.8–4.5) [79]. It is noteworthy that the reproduction number of HAV is of the same order of magnitude of the R_e_ values of sexually transmissible infection, such as HIV and HSV-2, in MSM population with long-term partnerships (1–2 years) [80].

The present study deeply explored the phylogenetic dynamics of the HAV outbreak occurred in a restricted geographical region of Italy during 2016–2017, mainly affecting MSM, where two monophyletic strains strongly predominated. The analysis HAV dataset in Lazio showed an epidemic continuity with the continental outbreak in MSM population across Europe. In addition, the origin of the strains was dated back to 2012, a date consistent with a silent circulation of these strains for a while, followed by explosive expansion, likely begun by the 2016 EuroPride held in Amsterdam, and an exportation across Europe and to other more distant countries.

Timely and regular monitoring of heterogeneity of virus population circulating in a given territory, using also public web-based phylodynamic tools updated in real-time (i.e. nextstrain.org), may represent a valuable tool for early appreciation of an impending epidemic event. More accurate standardization of parameters and thresholds to be considered as triggers of epidemic risk is needed, and to activate appropriate countermeasures and instrumental to promote targeted campaigns of vaccination.

In conclusion, this study opens the possibility to inform in real-time public health stakeholder policies and to explore though simulations the behaviour of different models.

## Supporting information

S1 Fig Phylogeography of all HAV strains. Panel A shows the phylogenetic tree, based on VP1X2A junction sequences of HAV genome, as obtained by Nextstrain, labelled by country. The sequences from Lazio, Italy are coloured in light green.
(TIFF)

S2 Fig Bayesian skyline plot of HAV genotype IA database. The demographic history of genotype IA, based on unique VP1X2A junction sequences of HAV genome, was inferred by a Bayesian skyline plot (BSP). The y-axis reports virus effective population size (Ne), the effective number of viral genomes contributing to new infections, while the x-axis is time in calendar years. Blue line is median estimated, while light blue area is 95% highest posterior density intervals (HPD).
(TIFF)

S3 Fig Maximum-likelihood phylogenetic analysis of all HAV sequences from Lazio region, Italy, and from Barcelona, Spain. Phylogenetic Maximum-Likelihood tree, built with a total of 246 HAV sequences from Lazio region, Italy, and 17 HAV sequences from Barcelona, Spain [55] (MK116906 to MK116923), using only 316 nt-long sequences of VP1X2A junction region of HAV genome. The tree was based on the maximum-likelihood method with the General Time Reversible model + G. All the sequences obtained until October 2018 from Lazio region are included. Moreover, the tree includes 16 reference sequences from GenBank (genotype IA: EU131373; AB020565.1; genotype IB: M14707; DQ646426; NC001489; AF314208; genotype IIA: AY644676; genotype IIB: AY644670; genotype IIIA: AJ299464; DQ991030; AB279733; genotype IIIB: AB279735; AB425339; AB258387), and four sequences (VRD_521_2016 and RIVM-HAV16-90, RIVM-HAV16-69 and V16_25801, in blue) associated with epidemic clusters among MSM. The bar represents the genetic distance (substitution per nucleotide position). Bootstrap analysis with 10,000 replicates was performed to assess the significance of the nodes; values greater than 80.
(TIFF)

S1 Table List of HAV strains collected in Lazio, Italy. Absolute frequencies and percentage (in brackets) of HAV unique based on VP1X2A junction variant sequences (strains), found in each annual viral population sample in Lazio.
(DOCX)

S2 Table HAV database used for Nextstrain tree. List of HAV sequences and information, using for interactive phylodynamic analysis on Nextstrain.org.
(DOCX)
