# Supplementary figures and images for: Origin of HAV strains responsible for 2016–2017 outbreak among MSM: Viral phylodynamics in Lazio region
Source: PLoS One. 2020 May 29;15(5):e0234010. doi: 10.1371/journal.pone.0234010 (PMC7259881; doi:10.1371/journal.pone.0234010)

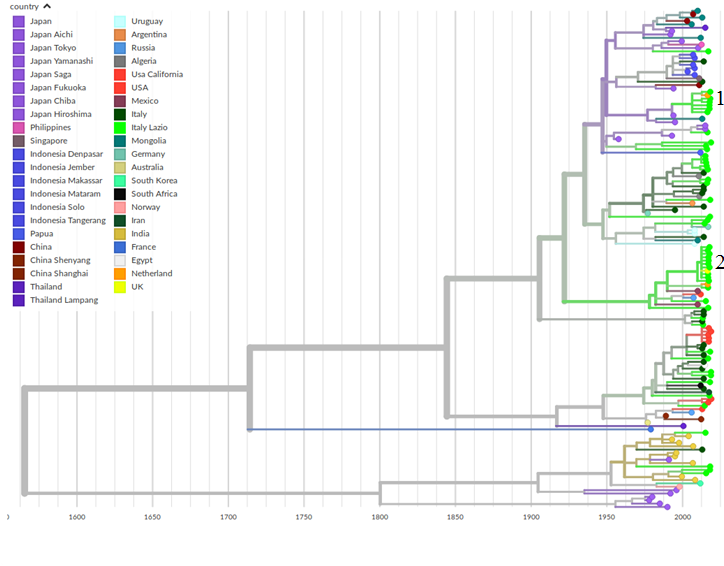

Supplement: S1 Fig — Panel A shows the phylogenetic tree, based on VP1X2A junction sequences of HAV genome, as obtained by Nextstrain, labelled by country. The sequences from Lazio, Italy are coloured in light green. (TIFF) [file pone.0234010.s001.tiff]

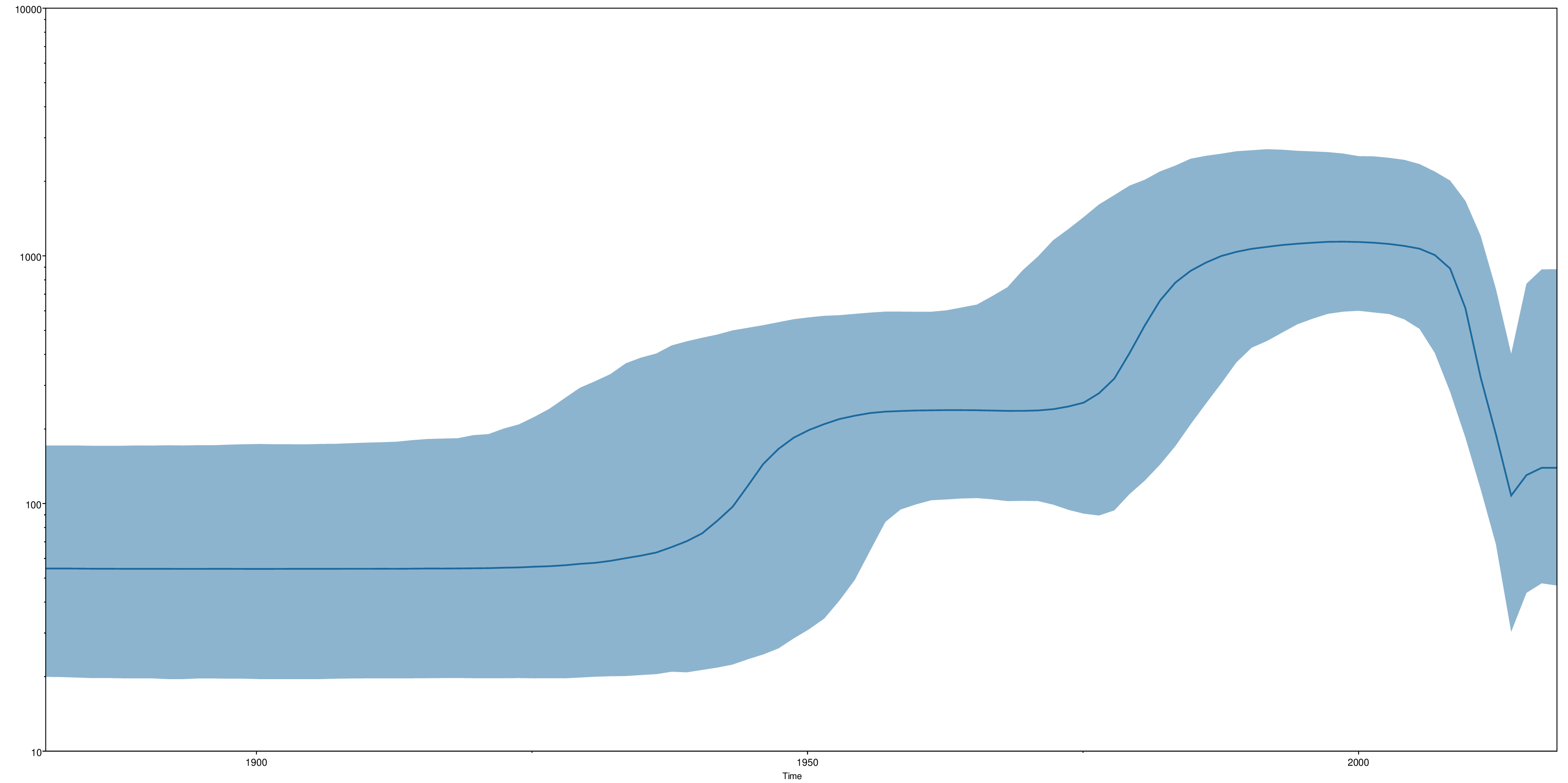

Supplement: S2 Fig — The demographic history of genotype IA, based on unique VP1X2A junction sequences of HAV genome, was inferred by a Bayesian skyline plot (BSP). The y-axis reports virus effective population size (Ne), the effective number of viral genomes contributing to new infections, while the x-axis is time in calendar years. Blue line is median estimated, while light blue area is 95% highest posterior density intervals (HPD). (TIFF) [file pone.0234010.s002.tiff]

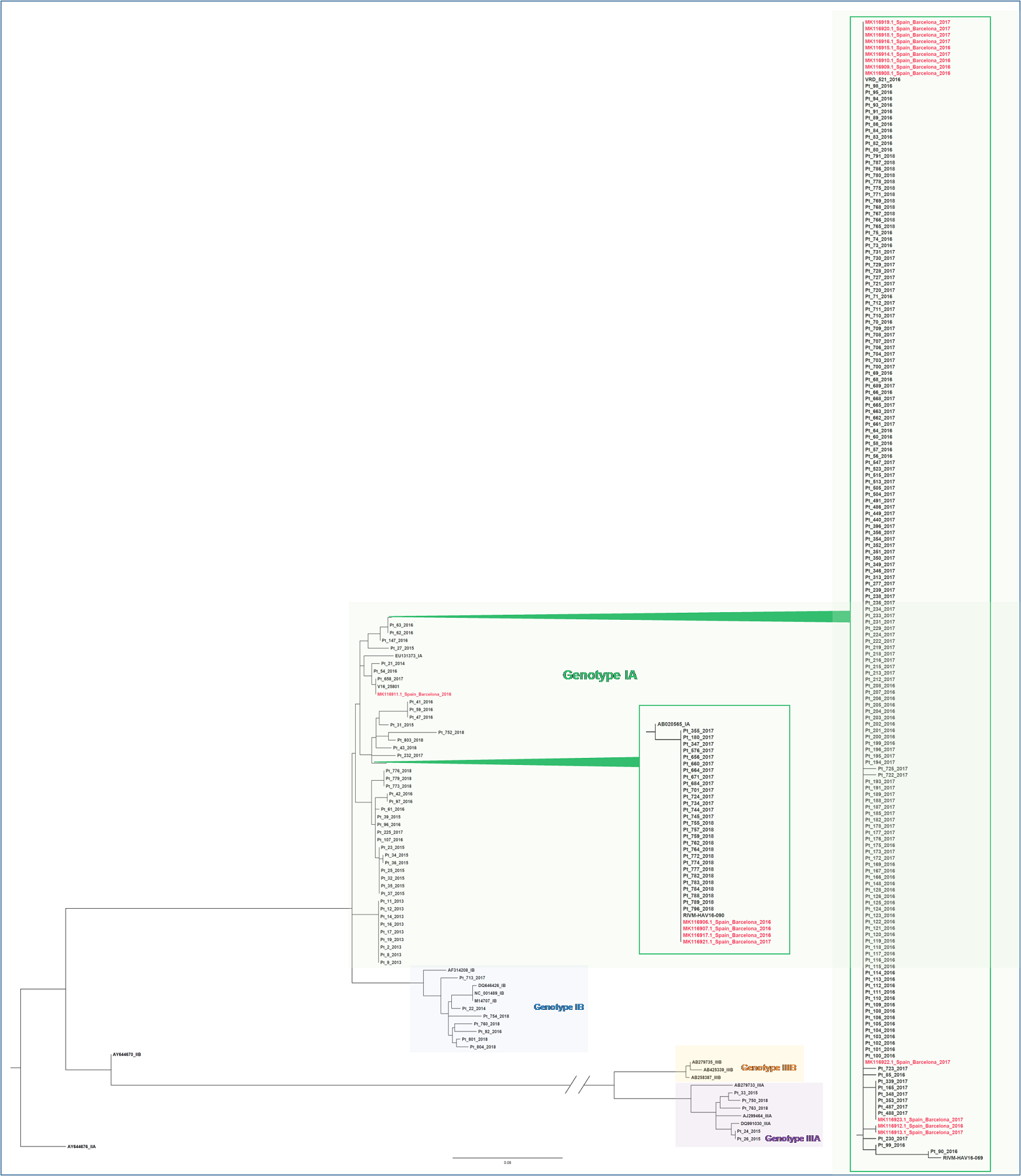

Supplement: S3 Fig — Phylogenetic Maximum-Likelihood tree, built with a total of 246 HAV sequences from Lazio region, Italy, and 17 HAV sequences from Barcelona, Spain [55] (MK116906 to MK116923), using only 316 nt-long sequences of VP1X2A junction region of HAV genome. The tree was based on the maximum-likelihood method with the General Time Reversible model + G. All the sequences obtained until October 2018 from Lazio region are included. Moreover, the tree includes 16 reference sequences from GenBank (genotype IA: EU131373; AB020565.1; genotype IB: M14707; DQ646426; NC001489; AF314208; genotype IIA: AY644676; genotype IIB: AY644670; genotype IIIA: AJ299464; DQ991030; AB279733; genotype IIIB: AB279735; AB425339; AB258387), and four sequences (VRD_521_2016 and RIVM-HAV16-90, RIVM-HAV16-69 and V16_25801, in blue) associated with epidemic clusters among MSM. The bar represents the genetic distance (substitution per nucleotide position). Bootstrap analysis with 10,000 replicates was performed to assess the significance of the nodes; values greater than 80. (TIFF) [file pone.0234010.s003.tiff]
