# Supplementary material for: Origin of HAV strains responsible for 2016–2017 outbreak among MSM: Viral phylodynamics in Lazio region
Source: PLoS One. 2020 May 29;15(5):e0234010. doi: 10.1371/journal.pone.0234010 (PMC7259881; doi:10.1371/journal.pone.0234010)
Supplement: S1 Table — Absolute frequencies and percentage (in brackets) of HAV unique based on VP1X2A junction variant sequences (strains), found in each annual viral population sample in Lazio. (DOCX) [file pone.0234010.s004.docx]

**S1 Table. List of HAV strains collected in Lazio, Italy.** Absolute frequencies and percentage (in brackets) of HAV unique based on VP1X2A junction variant sequences (strains), found in each annual viral population sample in Lazio.

| **ID_Pt** | **Genotype** | **Accession number** | **Collection date** | 2013-2015 | 2016 | 2017 | 2018 |
| --- | --- | --- | --- | --- | --- | --- | --- |
| **Pt_14_2013** | **IA** | **KY292294** | 13/07/2013 | 1 (4) | 0 (0) | 0 (0) | 0 (0) |
| **Pt_147_2016** | **IA** | **MH271362** | 29/12/2016 | 0 (0) | 1 (1) | 0 (0) | 0 (0) |
| **Pt_165_2017** | **IA** | **MH271363** | 04/01/2017 | 0 (0) | 0 (0) | 6 (6) | 0 (0) |
| **Pt_21_2014** | **IA** | **KY292305** | 01/08/2014 | 1 (4) | 0 (0) | 0 (0) | 0 (0) |
| **Pt_22_2014** | **IB** | **KY292307** | 20/08/2014 | 1 (4) | 0 (0) | 0 (0) | 0 (0) |
| **Pt_23_2015** | **IA** | **KY292303** | 05/02/2015 | 1 (4) | 0 (0) | 0 (0) | 0 (0) |
| **Pt_230_2017** | **IA** | **MK107986** | 31/01/2017 | 0 (0) | 0 (0) | 1 (1) | 0 (0) |
| **Pt_232_2017** | **IA** | **MH271365** | 31/01/2017 | 0 (0) | 0 (0) | 1 (1) | 0 (0) |
| **Pt_26_2015** | **IIIA** | **KY292308** | 18/02/2015 | 2 (8) | 0 (0) | 0 (0) | 0 (0) |
| **Pt_27_2015** | **IA** | **KY292293** | 22/05/2015 | 1 (4) | 0 (0) | 0 (0) | 0 (0) |
| **Pt_31_2015** | **IA** | **KY292297** | 22/06/2015 | 1 (4) | 0 (0) | 0 (0) | 0 (0) |
| **Pt_33_2015** | **IIIA** | **KY292309** | 23/07/2015 | 1 (4) | 0 (0) | 0 (0) | 0 (0) |
| **Pt_36_2015** | **IA** | **KY292302** | 26/07/2015 | 2 (8) | 0 (0) | 0 (0) | 0 (0) |
| **Pt_37_2015** | **IA** | **KY292303** | 05/02/2015 | 4 (17) | 0 (0) | 0 (0) | 0 (0) |
| **Pt_39_2015** | **IA** | **KY292301** | 07/12/2015 | 1 (4) | 2 (2) | 1 (1) | 0 (0) |
| **Pt_396_2017** | **IA** | **MK107987** | 27/03/2017 | 0 (0) | 0 (0) | 1 (1) | 0 (0) |
| **Pt_42_2016** | **IA** | **KY292299** | 21/03/2016 | 0 (0) | 1 (1) | 0 (0) | 0 (0) |
| **Pt_43_2016** | **IA** | **KY292296** | 13/04/2016 | 0 (0) | 1 (1) | 0 (0) | 0 (0) |
| **Pt_54_2016** | **IA** | **KY292304** | 07/08/2016 | 0 (0) | 1 (1) | 0 (0) | 0 (0) |
| **Pt_59_2016** | **IA** | **KY292298** | 15/03/2016 | 0 (0) | 3 (4) | 0 (0) | 0 (0) |
| **Pt_61_2016** | **IA** | **KY308187** | 13/09/2016 | 0 (0) | 1 (1) | 0 (0) | 0 (0) |
| **Pt_63_2016** | **IA** | **KY292291** | 15/09/2016 | 0 (0) | 2 (2) | 0 (0) | 0 (0) |
| **Pt_660_2017** | **IA** | **MH271367** | 22/06/2017 | 0 (0) | 0 (0) | 2 (2) | 0 (0) |
| **Pt_664_2017** | **IA** | **MH271369** | 29/09/2017 | 0 (0) | 0 (0) | 1 (1) | 0 (0) |
| **Pt_668_2017** | **IA** | **MH271366** | 13/05/2017 | 0 (0) | 0 (0) | 2 (2) | 0 (0) |
| **Pt_713_2017** | **IB** | **MH271370** | 27/09/2017 | 0 (0) | 0 (0) | 1 (1) | 0 (0) |
| **Pt_722_2017** | **IA** | **MK107988** | 18/10/2017 | 0 (0) | 0 (0) | 1 (1) | 0 (0) |
| **Pt_723_2017** | **IA** | **MK107989** | 18/10/2017 | 0 (0) | 0 (0) | 1 (1) | 0 (0) |
| **Pt_725_2017** | **IA** | **MK107990** | 18/10/2017 | 0 (0) | 0 (0) | 1 (1) | 0 (0) |
| **Pt_734_2017** | **IA** | **MH271371** | 19/10/2017 | 0 (0) | 0 (0) | 1 (1) | 0 (0) |
| **Pt_744_2017** | **IA** | **MH271372** | 24/11/2017 | 0 (0) | 0 (0) | 2 (2) | 3 (8) |
| **Pt_750_2018** | **IIIA** | **MH271373** | 10/02/2018 | 0 (0) | 0 (0) | 0 (0) | 1 (3) |
| **Pt_752_2018** | **IA** | **MH271374** | 16/02/2018 | 0 (0) | 0 (0) | 0 (0) | 1 (3) |
| **Pt_754_2018** | **IB** | **MH271375** | 23/02/2018 | 0 (0) | 0 (0) | 0 (0) | 1 (3) |
| **Pt_760_2018** | **IB** | **MH271376** | 13/03/2018 | 0 (0) | 0 (0) | 0 (0) | 1 (3) |
| **Pt_763_2018** | **IIIA** | **MK107993** | 19/03/2018 | 0 (0) | 0 (0) | 0 (0) | 1 (3) |
| **Pt_777_2018** | **IA** | **MK107991** | 13/04/2018 | 0 (0) | 0 (0) | 0 (0) | 1 (3) |
| **Pt_779_2018** | **IA** | **MH271377** | 31/03/2018 | 0 (0) | 0 (0) | 0 (0) | 3 (8) |
| **Pt_783_2018** | **IA** | **MK107992** | 13/04/2018 | 0 (0) | 0 (0) | 0 (0) | 2 (5) |
| **Pt_801_2018** | **IB** | **MK107994** | 02/10/2018 | 0 (0) | 0 (0) | 0 (0) | 1 (3) |
| **Pt_803_2018** | **IA** | **MK462239** | 16/10/2018 | 0 (0) | 0 (0) | 0 (0) | 1 (3) |
| **Pt_804_2018** | **IB** | **MK462240** | 20/10/2018 | 0 (0) | 0 (0) | 0 (0) | 1 (3) |
| **Pt_85_2016** | **IA** | **KY292290** | 04/11/2016 | 0 (0) | 1 (1) | 0 (0) | 0 (0) |
| **Pt_9_2013** | **IA** | **KY292295** | 02/06/2013 | 8 (33) | 0 (0) | 0 (0) | 0 (0) |
| **Pt_90_2016** | **IA** | **KY292292** | 11/11/2016 | 0 (0) | 1 (1) | 0 (0) | 0 (0) |
| **Pt_92_2016** | **IB** | **KY292306** | 11/11/2016 | 0 (0) | 1 (1) | 0 (0) | 0 (0) |
| **Pt_97_2016** | **IA** | **KY292300** | 16/11/2016 | 0 (0) | 1 (1) | 0 (0) | 0 (0) |
| **Pt_99_2016** | **IA** | **MH271361** | 22/11/2016 | 0 (0) | 1 (1) | 0 (0) | 0 (0) |
| **RIVM-HAV16-069** | **IA** |  |  | 0 (0) | 0 (0) | 0 (0) | 0 (0) |
| **RIVM-HAV16-090** | **IA** |  |  | 0 (0) | 0 (0) | 8 (8) | 8 (22) |
| **V16_2501** | **IA** |  |  | 0 (0) | 0 (0) | 1 (1) | 0 (0) |
| **VRD_521_2016** | **IA** |  |  | 0 (0) | 66 (80) | 70 (69) | 12 (32) |
|  |  |  |  | 24 (100) | 83 (100) | 101 (100) | 37 (100) |
