# Supplementary material for: Origin of HAV strains responsible for 2016–2017 outbreak among MSM: Viral phylodynamics in Lazio region
Source: PLoS One. 2020 May 29;15(5):e0234010. doi: 10.1371/journal.pone.0234010 (PMC7259881; doi:10.1371/journal.pone.0234010)
Supplement: S2 Table — List of HAV sequences and information, using for interactive phylodynamic analysis on Nextstrain.org. (DOCX) [file pone.0234010.s005.docx]

**S2 Table. HAV database used for Nextstrain tree.** List of HAV sequences and information, using for interactive phylodynamic analysis on Nextstrain.org.

| Strain | virus | date | authors | genotype | region | country | accession |
| --- | --- | --- | --- | --- | --- | --- | --- |
| AB020564.1_Japan_1992 | HAV | XX-XX-1992 | Fujiwara et al., 2001 | IA | asia | japan | AB020564.1 |
| AB020565.1_Japan_1993 | HAV | XX-XX-1993 | Fujiwara et al., 2001 | IA | asia | japan | AB020565.1 |
| AB020566.1_Japan_1994 | HAV | XX-XX-1994 | Fujiwara et al., 2001 | IA | asia | japan | AB020566.1 |
| AB020567.1_Japan_1992 | HAV | XX-XX-1992 | Fujiwara et al., 2001 | IA | asia | japan | AB020567.1 |
| AB020568.1_Japan_1991 | HAV | XX-XX-1991 | Fujiwara et al., 2001 | IA | asia | japan | AB020568.1 |
| AB020569.1_Japan_1993 | HAV | XX-XX-1993 | Fujiwara et al., 2001 | IA | asia | japan | AB020569.1 |
| AB258387.1_Japan_Aichi_1990 | HAV | XX-XX-1990 | Endo et a., 2007 | IIIB | asia | japan_Aichi | AB258387.1 |
| AB279732.1_Japan_Aichi_1990 | HAV | XX-XX-1990 | Endo et a., 2007 | IIIA | asia | japan_Aichi | AB279732.1 |
| AB279733.1_Japan_Aichi_1992 | HAV | XX-XX-1992 | Endo et a., 2007 | IIIA | asia | japan_Aichi | AB279733.1 |
| AB279734.1_Japan_Tokyo_1995 | HAV | XX-XX-1995 | Endo et a., 2007 | IIIA | asia | japan_Tokyo | AB279734.1 |
| AB279735.1_Japan_Yamanashi_1985 | HAV | XX-XX-1985 | Endo et a., 2007 | IIIB | asia | japan_Yamanashi | AB279735.1 |
| AB300205.1_Japan_Saga_1977 | HAV | XX-XX-1977 | Yoneyama et al., 2007 | IIIB | asia | japan_Saga | AB300205.1 |
| AB425339.1_Japan_Fukuoka_1979 | HAV | XX-XX-1979 | Shimasaki et al., unp. | IIIB | asia | japan_Fukuoka | AB425339.1 |
| AB618529.1_Japan_Fukuoka_2010 | HAV | 12-Apr-2010 | Dohmen et al., 2011 | IA | asia | japan_Fukuoka | AB618529.1 |
| AB618531.1_Japan_Hokkaido_Sapporo_1999 | HAV | 17-Jun-1999 | Dohmen et al., 2011 | IA | asia | japan | AB618531.1 |
| AB623053.1_Japan_Akita_Osarizawa_1957 | HAV | 4-Oct-1957 | Takahashi et al 2011 | IA | asia | japan | AB623053.1 |
| AB793726.1_Japan_Philippines_2012 | HAV | 23-Jul-2012 | Watanabe et al 2014 | IA | asia | philippines | AB793726.1 |
| AB819869.1_Singapore_2011 | HAV | XX-XX-2011 | Mishiro et al | IA | asia | singapore | AB819869.1 |
| AB839692.1_Indonesia_Bali(Denpasar)_2003 | HAV | XX-XX-2003 | Mulyanto et al 2014 | IA | asia | indonesia_Denpasar | AB839692.1 |
| AB839693.1_Indonesia_EastJava(Jember)_2007 | HAV | XX-XX-2007 | Mulyanto et al 2014 | IA | asia | indonesia_Jember | AB839693.1 |
| AB839694.1_Indonesia_Sulawesi(Makassar)_2007 | HAV | XX-XX-2007 | Mulyanto et al 2014 | IA | asia | indonesia_Makassar | AB839694.1 |
| AB839695.1_Indonesia_Lombok(Mataram)_2007 | HAV | XX-XX-2007 | Mulyanto et al 2014 | IA | asia | indonesia_Mataram | AB839695.1 |
| AB839696.1_Indonesia_CentralJava(Solo)_2007 | HAV | XX-XX-2007 | Mulyanto et al 2014 | IA | asia | indonesia_Solo | AB839696.1 |
| AB839697.1_Indonesia_WestJava(Tangerang)_2007 | HAV | XX-XX-2007 | Mulyanto et al 2014 | IA | asia | indonesia_Tangerang | AB839697.1 |
| AB909123.1_Japan_Tokyo_Papua_2011 | HAV | 18-Oct-2011 | Sugihara et al | IA | asia | papua | AB909123.1 |
| AF485328.1_China_2001 | HAV | XX-XX-2011 | Hu et al | IA | asia | china | AF485328.1 |
| AF512536.1_China_2003 | HAV | XX-XX-2003 | Liu et al | IA | asia | china | AF512536.1 |
| AJ299464.1_Norway_Oestlandet_1997 | HAV | XX-XX-1997 | Stene-Johansen et al., 2005 | IIIA | europe | norway | AJ299464.1 |
| AY644670.1_SierraLeone_1988 | HAV | XX-XX-1988 | Ching et al., 2002 | IIB | africa | sierra_leone | AY644670.1 |
| AY644676.1_France_1979 | HAV | XX-XX-1979 | Lu et a., 2004 | IIA | europe | france | AY644676.1 |
| DQ646426.1_Russia_2006 | HAV | XX-XX-2006 | Bitchenco et al. | IB | europe | russia | DQ646426.1 |
| DQ991029.1_India_1995 | HAV | XX-XX-1995 | Joshi et al., 2010 | IIIA | asia | india | DQ991029.1 |
| DQ991030.1_India_2003 | HAV | XX-XX-2003 | Joshi et al., 2010 | IIIA | asia | india | DQ991030.1 |
| EF207320.1_Thailand_Lampang_2014 | HAV | XX-XX-2014 | Barameechai et al | IA | asia | thailand_Lampang | EF207320.1 |
| EU011791.1_India_1992 | HAV | XX-XX-1992 | Joshi et al., 2008 | IIIA | asia | india | EU011791.1 |
| EU131373.1_Uruguay_2007 | HAV | XX-XX-2007 | Garcia-Aguirre et al 2008 | IA | south_america | uruguay | EU131373.1 |
| EU526088.1_Uruguay_2008 | HAV | XX-XX-2008 | Garcia-Aguirre et al | IA | south_america | uruguay | EU526088.1 |
| EU526089.1_Uruguay_2008 | HAV | XX-XX-2008 | Garcia-Aguirre et al | IA | south_america | uruguay | EU526089.1 |
| FJ360730.1_India_2006 | HAV | 13-Oct-2006 | Kulkarni et al., 2009 | IIIA | asia | india | FJ360730.1 |
| FJ360731.1_India_2007 | HAV | XX-XX-2007 | Kulkarni et al., 2009 | IIIA | asia | india | FJ360731.1 |
| FJ360732.1_India_2008 | HAV | 11-Apr-2008 | Kulkarni et al., 2009 | IIIA | asia | india | FJ360732.1 |
| FJ360733.1_India_1995 | HAV | 29-Nov-1995 | Kulkarni et al., 2009 | IIIA | asia | india | FJ360733.1 |
| FJ360734.1_India_1999 | HAV | 20-Jul-1999 | Kulkarni et al., 2009 | IIIA | asia | india | FJ360734.1 |
| FJ360735.1_India_1997 | HAV | 29-Aug-1997 | Kulkarni et al., 2009 | IIIA | asia | india | FJ360735.1 |
| HM769724.1_Argentina_2006 | HAV | 11-Jun-2006 | Aguirre et al 2011 | IA | south_america | argentina | HM769724.1 |
| HQ437707.1_Russia_2007 | HAV | 10-Apr-2007 | Bondarenko et al | IA | europe | russia | HQ437707.1 |
| JN873911.1_Algeria_2010 | HAV | XX-Oct-2007 | Boxman et al 2012 | IA | africa | algeria | JN873911.1 |
| JN873912.1_Algeria_2011 | HAV | XX-Feb-2011 | Boxman et al 2012 | IA | africa | algeria | JN873912.1 |
| JQ425480.1_USA_2012 | HAV | XX-XX-2012 | Chizhikov, V. | IA | north_america | usa | JQ425480.1 |
| JQ655151.1_SouthKorea_2011 | HAV | 1-Oct-2011 | Lee et al., 2013 | IIIA | asia | south_korea | JQ655151.1 |
| KC182588.1_Mexico_2009 | HAV | XX-XX-2009 | Vaughan et al 2014 | IA | north_america | mexico | KC182588.1 |
| KC182590.1_Mexico_2009 | HAV | XX-XX-2009 | Vaughan et al 2014 | IA | north_america | mexico | KC182590.1 |
| KF569906.1_China_LiaoningProvince_ShenyangCity_2012 | HAV | 18-Apr-2012 | Zhou et al., unp. | IB | asia | china_Shenyang | KF569906.1 |
| KP177964.1_China_2007 | HAV | XX-XX-2007 | Wang et al | IA | asia | china | KP177964.1 |
| KU570221.1_Italy_2013 | HAV | XX-XX-2013 | Bruni et al 2016 | IA | europe | italy | KU570221.1 |
| KU570227.1_Italy_2013 | HAV | XX-XX-2013 | Bruni et al 2016 | IA | europe | italy | KU570227.1 |
| KU570229.1_Italy_2013 | HAV | XX-XX-2013 | Bruni et al 2016 | IA | europe | italy | KU570229.1 |
| KU570232.1_Italy_2013 | HAV | XX-XX-2013 | Bruni et al 2016 | IA | europe | italy | KU570232.1 |
| KU570236.1_Italy_2013 | HAV | XX-XX-2013 | Bruni et al 2016 | IA | europe | italy | KU570236.1 |
| KU570239.1_Italy_2013 | HAV | XX-XX-2013 | Bruni et al 2016 | IA | europe | italy | KU570239.1 |
| KU570243.1_Italy_2013 | HAV | XX-XX-2013 | Bruni et al 2016 | IB | europe | italy | KU570243.1 |
| KU570244.1_Italy_2013 | HAV | XX-XX-2013 | Bruni et al 2016 | IB | europe | italy | KU570244.1 |
| KU570245.1_Italy_2013 | HAV | XX-XX-2013 | Bruni et al 2016 | IB | europe | italy | KU570245.1 |
| KU570246.1_Italy_2013 | HAV | XX-XX-2013 | Bruni et al 2016 | IB | europe | italy | KU570246.1 |
| KU570247.1_Italy_2013 | HAV | XX-XX-2013 | Bruni et al 2016 | IB | europe | italy | KU570247.1 |
| KU570251.1_Italy_2013 | HAV | XX-XX-2013 | Bruni et al 2016 | IA | europe | italy | KU570251.1 |
| KU570282.1_Italy_2013 | HAV | XX-XX-2013 | Bruni et al 2016 | IA | europe | italy | KU570282.1 |
| KU570284.1_Italy_2013 | HAV | XX-XX-2013 | Bruni et al 2016 | IA | europe | italy | KU570284.1 |
| KU570285.1_Italy_2013 | HAV | XX-XX-2013 | Bruni et al 2016 | IA | europe | italy | KU570285.1 |
| KU570289.1_Italy_2013 | HAV | XX-XX-2013 | Bruni et al 2016 | IB | europe | italy | KU570289.1 |
| KU570290.1_Italy_2013 | HAV | XX-XX-2013 | Bruni et al 2016 | IA | europe | italy | KU570290.1 |
| KU570291.1_Italy_2013 | HAV | XX-XX-2013 | Bruni et al 2016 | IB | europe | italy | KU570291.1 |
| KU570292.1_Italy_2013 | HAV | XX-XX-2013 | Bruni et al 2016 | IIIA | europe | italy | KU570292.1 |
| KX035096.1_USA_2013 | HAV | XX-XX-2013 | Yang et al. | IB | north_america | usa | KX035096.1 |
| KX228694.1_Egypt_2014 | HAV | XX-XX-2014 | Hamza et al., 2017 | IB | africa | egypt | KX228694.1 |
| KX523680.1_China_Shanghai_1988 | HAV | XX-XX-1988 | Jiang et al., unp | IB | asia | china_Shanghai | KX523680.1 |
| KY003229.1_SouthAfrica_2011 | HAV | XX-XX-2011 | Gall et al. Unp | IB | asia | south_africa | KY003229.1 |
| LC049337.1_Mongolia_Ulaanbaatar_2012 | HAV | XX-XX-2012 | Tsatsralt-Od et al 2016 | IA | asia | mongolia | LC049337.1 |
| LC049338.1_Mongolia_Ulaanbaatar_2009 | HAV | XX-XX-2009 | Tsatsralt-Od et al 2016 | IA | asia | mongolia | LC049338.1 |
| LC049339.1_Mongolia_Ulaanbaatar_2006 | HAV | XX-XX-2006 | Tsatsralt-Od et al 2016 | IA | asia | mongolia | LC049339.1 |
| LC049340.1_Mongolia_Ulaanbaatar_2010 | HAV | XX-XX-2010 | Tsatsralt-Od et al 2016 | IA | asia | mongolia | LC049340.1 |
| LC049341.1_Mongolia_Ulaanbaatar_2012 | HAV | XX-XX-2012 | Tsatsralt-Od et al 2016 | IA | asia | mongolia | LC049341.1 |
| LC049342.1_Mongolia_Ulaanbaatar_2012 | HAV | XX-XX-2013 | Tsatsralt-Od et al 2016 | IA | asia | mongolia | LC049342.1 |
| LC128713.1_Thailand_2000 | HAV | XX-Aug-2000 | Ishii unp. | IB | asia | thailand | LC128713.1 |
| LC191189.1_Japan_Chiba_2015 | HAV | XX-XX-2015 | Miura et al 2017 | IA | asia | japan_Chiba | LC191189.1 |
| LC373510.1_Japan_Hiroshima_2014 | HAV | XX-XX-2014 | Saito et al 2018 | IA | asia | japan_Hiroshima | LC373510.1 |
| M14707.1_Australia_1976 | HAV | XX-XX-1976 | Cohen et al 1987 | IB | oceania | australia | M14707.1 |
| MG546668.1_Iran_Ahvaz_2017 | HAV | 22-Jun-2017 | Nejati et al., unp. | IB | asia | iran | MG546668.1 |
| MH577308.1_USA_2017 | HAV | XX-XX-2017 | Probert et al., unp | IB | north_america | usa_california | MH577308.1 |
| MH577309.1_USA_2017 | HAV | XX-XX-2017 | Probert et al., unp | IB | north_america | usa_california | MH577309.1 |
| MH577310.1_USA_2017 | HAV | XX-XX-2017 | Probert et al., unp | IB | north_america | usa_california | MH577310.1 |
| MH577311.1_USA_2018 | HAV | XX-XX-2018 | Probert et al., unp | IB | north_america | usa_california | MH577311.1 |
| MH577312.1_USA_2017 | HAV | XX-XX-2017 | Probert et al., unp | IB | north_america | usa_california | MH577312.1 |
| MH577313.1_USA_2017 | HAV | XX-XX-2017 | Probert et al., unp | IB | north_america | usa_california | MH577313.1 |
| MH577314.1_USA_2018 | HAV | XX-XX-2018 | Probert et al., unp | IB | north_america | usa_california | MH577314.1 |
| Pt_14_2013 | HAV | 13-Jul-2013 | Minosse et al. | IA | europe | italy_Lazio | KY292294 |
| Pt_147_2016 | HAV | 16-Dec-2016 | Lanini et al. 2016 | IA | europe | italy_Lazio | MH271362 |
| Pt_165_2017 | HAV | 4-Jan-2017 | Lanini et al. 2016 | IA | europe | italy_Lazio | MH271363 |
| Pt_21_2014 | HAV | 1-Aug-2014 | Minosse et al. | IA | europe | italy_Lazio | KY292305 |
| Pt_22_2014 | HAV | 20-Aug-2014 | Minosse et al. | IB | europe | italy_Lazio | KY292307 |
| Pt_23_2015 | HAV | 5-Feb-2015 | Minosse et al. | IA | europe | italy_Lazio | KY292303 |
| Pt_230_2017 | HAV | 31-Jan-2017 | Lanini et al. 2016 | IA | europe | italy_Lazio | MK107986 |
| Pt_232_2017 | HAV | 17-Jan-2017 | Lanini et al. 2016 | IA | europe | italy_Lazio | MH271365 |
| Pt_26_2015 | HAV | 18-Feb-2015 | Minosse et al. | IIIA | europe | italy_Lazio | KY292308 |
| Pt_27_2015 | HAV | 22-May-2015 | Minosse et al. | IA | europe | italy_Lazio | KY292293 |
| Pt_31_2015 | HAV | 22-Jun-2015 | Minosse et al. | IA | europe | italy_Lazio | KY292297 |
| Pt_33_2015 | HAV | 23-Jul-2015 | Minosse et al. | IIIA | europe | italy_Lazio | KY292309 |
| Pt_36_2015 | HAV | 26-Jul-2015 | Minosse et al. | IA | europe | italy_Lazio | KY292302 |
| Pt_37_2015 | HAV | 5-Feb-2015 | Minosse et al. | IA | europe | italy_Lazio | KY292303 |
| Pt_39_2015 | HAV | 7-Dec-2015 | Minosse et al. | IA | europe | italy_Lazio | KY292301 |
| Pt_396_2017 | HAV | 27-Mar-2017 | Lanini et al. 2016 | IA | europe | italy_Lazio | MK107987 |
| Pt_42_2016 | HAV | 21-Mar-2016 | Minosse et al. | IA | europe | italy_Lazio | KY292299 |
| Pt_43_2016 | HAV | 13-Jun-2016 | Lanini et al. 2016 | IA | europe | italy_Lazio | KY292296 |
| Pt_54_2016 | HAV | 7-Aug-2016 | Lanini et al. 2016 | IA | europe | italy_Lazio | KY292304 |
| Pt_59_2016 | HAV | 15-Mar-2016 | Minosse et al. | IA | europe | italy_Lazio | KY292298 |
| Pt_61_2016 | HAV | 13-Sep-2016 | Lanini et al. 2016 | IA | europe | italy_Lazio | KY308187 |
| Pt_63_2016 | HAV | 15-Sep-2016 | Lanini et al. 2016 | IA | europe | italy_Lazio | KY292291 |
| Pt_660_2017 | HAV | 17-Jun-2016 | Lanini et al. 2016 | IA | europe | italy_Lazio | MH271367 |
| Pt_664_2017 | HAV | 29-Sep-2017 | Minosse et al. | IA | europe | italy_Lazio | MH271369 |
| Pt_668_2017 | HAV | 13-May-2017 | Lanini et al. 2016 | IA | europe | italy_Lazio | MH271366 |
| Pt_713_2017 | HAV | 27-Sep-2017 | Minosse et al. | IB | europe | italy_Lazio | MH271370 |
| Pt_722_2017 | HAV | 18-Oct-2017 | Minosse et al. | IA | europe | italy_Lazio | MK107988 |
| Pt_723_2017 | HAV | 18-Oct-2017 | Minosse et al. | IA | europe | italy_Lazio | MK107989 |
| Pt_725_2017 | HAV | 17-Oct-2017 | Minosse et al. | IA | europe | italy_Lazio | MK107990 |
| Pt_734_2017 | HAV | 17-Oct-2017 | Minosse et al. | IA | europe | italy_Lazio | MH271371 |
| Pt_744_2017 | HAV | 24-Nov-2017 | Minosse et al. | IA | europe | italy_Lazio | MH271372 |
| Pt_750_2018 | HAV | 10-Feb-2018 | Minosse et al. | IIIA | europe | italy_Lazio | MH271373 |
| Pt_752_2018 | HAV | 16-Feb-2018 | Minosse et al. | IA | europe | italy_Lazio | MH271374 |
| Pt_754_2018 | HAV | 23-Feb-2018 | Minosse et al. | IB | europe | italy_Lazio | MH271375 |
| Pt_760_2018 | HAV | 13-Mar-2018 | Minosse et al. | IB | europe | italy_Lazio | MH271376 |
| Pt_763_2018 | HAV | 19-Mar-2018 | Minosse et al. | IIIA | europe | italy_Lazio | MK107993 |
| Pt_777_2018 | HAV | 13-Apr-2018 | Minosse et al. | IA | europe | italy_Lazio | MK107991 |
| Pt_779_2018 | HAV | 31-Mar-2018 | Minosse et al. | IA | europe | italy_Lazio | MH271377 |
| Pt_783_2018 | HAV | 13-Apr-2018 | Minosse et al. | IA | europe | italy_Lazio | MK107992 |
| Pt_801_2018 | HAV | 2-Oct-2018 | Minosse et al. | IB | europe | italy_Lazio | MK107994 |
| Pt_803_2018 | HAV | 16-Oct-2018 | Minosse et al. | IA | europe | italy_Lazio | MK462239 |
| Pt_804_2018 | HAV | 20-Oct-2018 | Minosse et al. | IB | europe | italy_Lazio | MK462240 |
| Pt_85_2016 | HAV | 4-Nov-2016 | Lanini et al. 2016 | IA | europe | italy_Lazio | KY292290 |
| Pt_9_2013 | HAV | 2-Jun-2013 | Minosse et al. | IA | europe | italy_Lazio | KY292295 |
| Pt_90_2016 | HAV | 11-Nov-2016 | Lanini et al. 2016 | IA | europe | italy_Lazio | KY292292 |
| Pt_92_2016 | HAV | 11-Nov-2016 | Lanini et al. 2016 | IB | europe | italy_Lazio | KY292306 |
| Pt_97_2016 | HAV | 16-Nov-2016 | Lanini et al. 2016 | IA | europe | italy_Lazio | KY292300 |
| Pt_99_2016 | HAV | 22-Nov-2016 | Lanini et al. 2016 | IA | europe | italy_Lazio | MH271361 |
| RIVM-HAV16-069 | HAV | XX-Aug-2000 | Freidl et al. 2017 | IA | europe | Netherland |  |
| RIVM-HAV16-090 | HAV | XX-Aug-2001 | Freidl et al. 2017 | IA | europe | Netherland |  |
| V16_25801 | HAV | XX-Aug-2002 | Werber et al. 2016 | IA | europe | germany |  |
| VRD_521_2016 | HAV | XX-Aug-2003 | Beebeejaun et al. 2017 | IA | europe | UK |  |
| X75215.1_Germany_1976 | HAV | XX-XX-1976 | Graff et al 1994 | IA | europe | germany | X75215.1 |
| X83302.1_Italy_1995 | HAV | XX-XX-1995 | Beneduce et al 1995 | IA | europe | italy | X83302.1 |
